# Morning Headaches as a Specific Obstructive Sleep Apnea Characteristic in a Large Sleep Clinic Population

**DOI:** 10.3390/life16081239

**Published:** 2026-07-27

**Authors:** Aliki Karkala, Alexandros Kalkanis, Serafeim-Chrysovalantis Kotoulas, Şakir Kaan Çetindağ, Dries Testelmans, Vasileios Paraschou, Dionisios Spyratos, Dimitrios G. Raptis, Paschalis Steiropoulos, Athanasia Pataka

**Affiliations:** 1 Center for Sleep and Wake Disorders (LUCS), University Hospitals Leuven Campus Gasthuisberg, Herestraat 49, 3000 Leuven, Belgium; aliki.karkala@uzleuven.be (A.K.); alexandros.kalkanis@uzleuven.be (A.K.); dries.testelmans@uzleuven.be (D.T.); 2 Laboratory of Respiratory Disease and Thoracic Surgery (BREATHE), KU Leuven-University, Herestraat 49, 3000 Leuven, Belgium; 3 Adult ICU, Ippokratio General Hospital of Thessaloniki, Konstantinoupoleos 49, 54642 Thessaloniki, Greece; akiskotoulas@hotmail.com; 4 Department of Biology, Leuven Brain Institute, KU Leuven, 3000 Leuven, Belgium; sakirkaan.cetindag@kuleuven.be; 5 Hellenic Police Medical Center, 54121 Thessaloniki, Greece; billisparas@gmail.com; 6 Pulmonary Department, Unit of Thoracic Malignancies Research, General Hospital of Thessaloniki “Georgios Papanikolaou”, Aristotle University of Thessaloniki, Leoforos Papanikolaou Municipality of Chortiatis, 57010 Thessaloniki, Greece; diospyrato@yahoo.gr; 7 Cardiology Department, General Hospital of Thessaloniki “Georgios Papanikolaou”, Leoforos Papanikolaou Municipality of Chortiatis, 57010 Thessaloniki, Greece; raptisdg@gmail.com; 8 Department of Respiratory Medicine, Medical School, Democritus University of Thrace, University General Hospital Dragana, 68100 Alexandroupolis, Greece; steiropoulos@yahoo.com; 9 Respiratory Failure Clinic and Sleep Laboratory, General Hospital of Thessaloniki “Georgios Papanikolaou”, Aristotle University of Thessaloniki, Leoforos Papanikolaou Municipality of Chortiatis, 57010 Thessaloniki, Greece

**Keywords:** morning headaches, obstructive sleep apnea, arousal index, sex differences

## Abstract

Morning headaches (MHs) are a recognized but incompletely understood symptom in obstructive sleep apnea (OSA). Because symptoms and comorbidities that commonly coexist with OSA may influence both headache susceptibility and perceived sleep quality, the respective contributions of respiratory abnormalities and sleep disruption to MHs remain uncertain, particularly in women. We therefore aimed to identify clinical and sleep study factors associated with frequent MHs in patients with OSA. In this retrospective cohort study, 3227 adults with confirmed OSA evaluated between 2013 and 2023 were classified according to MH frequency (<1 episode/week vs. ≥1 episode/week). Demographic, clinical, questionnaire and sleep study variables were analyzed using univariate and multivariable logistic regression. Frequent MHs were reported by 35.2% of patients. On univariate analysis, MHs were associated with female sex, higher body mass index and waist circumference, greater OSA severity, worse nocturnal oxygenation, fatigue, excessive daytime sleepiness, and insomnia symptoms. However, in multivariable analysis restricted to patients with complete polysomnographic data (N = 501), female sex (OR 4.23, 95% CI 1.39–12.85), waist circumference (OR 1.03, 95% CI 1.01–1.06), and arousal index (OR 1.05, 95% CI 1.01–1.08) emerged as independent predictors, whereas conventional respiratory severity indices did not. These findings suggest that sleep fragmentation, rather than OSA severity alone, may be a key determinant of MHs, particularly in women, underscoring the need for sex-specific approaches beyond apnea–hypopnea index-based assessment.

## 1. Introduction

Morning headaches (MHs), defined as cephalalgia occurring upon waking, affect 5–8% of the general population, with higher prevalence among women and individuals aged 45–64 years [1,2]. Diagnostic criteria remain heterogeneous across studies, with definitions including three or more MHs annually, weekly or daily occurrences, as well as qualitative descriptors such as “often” or “sometimes” [1,3,4]. The absence of standardized diagnostic criteria complicates epidemiological comparisons and highlights the need for nuanced clinical evaluation [5]. MHs are non-specific and of multifaceted etiology, encompassing both primary and secondary causes that intersect with physiological, behavioral and systemic mechanisms [5,6,7].

MHs represent a prevalent complaint among individuals with obstructive sleep apnea (OSA), a disease affecting 54% of the population [8], occurring at significantly higher rates compared to the general population without the disorder [1], but heterogeneity is high among studies [4]. Older studies report rates as high as 50% [9,10,11]. This heterogeneity is likely attributable to methodological differences, including retrospective versus prospective study designs, inconsistencies in defining headache and variations in ascertaining headache onset. Notably, headache frequency appears higher in OSA patients compared to individuals with other sleep disorders such as insomnia, snoring, or limb movement disorders [12]. In comparison, the prevalence of primary headache disorders like migraine and tension-type headache within OSA populations mirrors or is slightly above rates observed in the general population [4].

The clinical significance of headache as a manifestation of OSA is underscored by its inclusion in the International Classification of Headache Disorders (ICHD) in 2004, when a distinct diagnostic category for “sleep apnea headache” had been established [13]. As defined by the current ICHD-3 criteria, this specific headache type is characterized as a usually bilateral, pressing pain that occurs 15 or more days per month, typically resolving within 4 h of awakening and lacking accompanying symptoms [14]. Clinically, however, a broader presentation of dull, diffuse-pain MHs are also frequently observed upon waking in OSA patients, but these headaches may persist beyond 4 h and can be associated with additional symptomatology such as fatigue and nausea [15,16]. Research has predominantly focused on the role of nocturnal respiratory disturbances characteristic of OSA, including heavy snoring, intermittent hypoxia, hypercapnia and consequent blood pressure fluctuations, in provoking headaches that persist upon awakening [2]. Crucially, the consistent observation that effective OSA treatment, particularly CPAP therapy, resolves or significantly ameliorates MHs in affected patients [17,18], has provided compelling evidence for a causal relationship between OSA pathophysiology and headache generation [3,19]. Nevertheless, the ICHD-3 acknowledges persistent uncertainty regarding whether hypoxia, hypercapnia, sleep disturbance, or a combination thereof constitutes the primary driver of sleep apnea headache [14].

Furthermore, it is unclear how OSA severity relates to MHs [20,21], as they have demonstrated inconsistent correlations with standard metrics of OSA severity like the AHI or oxygen desaturation indices [2,22,23]. Emerging evidence suggests roles for REM-specific disturbances and epigenetic dysregulation, but conclusive mechanisms are lacking [24]. This ambiguity extends to treatment, where, although CPAP resolves MHs in most cases [17], a significant minority of non-responders suggests potential overlap with primary headache disorders or non-hypoxic mechanisms [25]. Consequently, it remains unclear whether MHs constitute a specific OSA phenotype linked to distinct mechanisms or merely a nonspecific symptom, particularly within diverse clinical populations.

Therefore, MH pathogenesis seems to be of a multifactorial nature [4], implying hypoxemia may act as a contributory factor primarily in susceptible individuals rather than a universal mechanism [2,21]. This susceptibility hypothesis theoretically aligns with the observed sex disparity in MH prevalence, where women with OSA are significantly more likely to report MHs alongside other non-classical symptoms, such as fatigue and insomnia, which may contribute to headache pathophysiology [26]. While women may experience heightened sensitivity to fatigue and sleep fragmentation that correlate with MHs but may not fully align with sleep study metrics like the AHI [24], the most recent meta-analytic data showed no significant sex effect on MH frequency [4]. Especially as few studies have systematically adjusted for potential confounding factors such as comorbidities, insomnia and somatometric characteristics, the absence of consistent sex differences may indicate that factors beyond OSA severity or biological sex itself drive the occurrence of MHs in OSA populations.

Indeed, symptoms that frequently coexist with OSA, including insomnia, snoring, mood disturbances, obesity-related phenotypes and cardiometabolic comorbidities, may influence both headache susceptibility and the subjective perception of sleep quality [7,26,27,28], therefore obscuring direct associations between respiratory indices and MHs. Consequently, the extent to which MHs represent a distinct manifestation of OSA, rather than a multifactorial symptom emerging from the interaction of sleep disruption, comorbidity burden and patient-specific vulnerability, remains insufficiently understood. This uncertainty is particularly relevant in women with OSA, who often present with atypical symptom profiles and a greater burden of non-restorative sleep complaints [29,30]. Therefore, the present cohort study aimed to identify clinical, besides sleep-study-derived, predictors of MHs in patients with OSA, with a particular focus on factors that may contribute to the occurrence of MHs in women.

## 2. Materials and Methods

This retrospective cohort study analyzed data from patients referred to the sleep clinic of the Respiratory Failure Unit, Department of Pulmonology, “Georgios Papanikolaou” General Hospital, Thessaloniki, Greece, between 2013 and 2023 for suspected OSA, based on symptoms including unrefreshing sleep, snoring and excessive daytime sleepiness (EDS).

During the initial outpatient visit at the sleep clinic, patients were interviewed regarding comorbidities, occurrence (yes/no) of sleep-related complaints and subjective daytime symptoms (e.g., fatigue, memory loss). Anthropometric characteristics (body measurements, height, weight and body mass index [BMI]) and demographic factors (sex, age) were also recorded. During the same visit, patients completed standardized questionnaires in Greek, including a sleep habits questionnaire, the Epworth Sleepiness Scale (ESS) to assess daytime sleepiness [31,32] and the Athens Insomnia Scale (AIS) to quantify the severity of any insomnia symptoms [33,34], also as part of the standardized routine of the sleep clinic. The sleep habits questionnaire assessed MH frequency at this initial visit, defined as headache upon awakening, with improvement occurring within hours. This questionnaire asked participants to report MH frequency using five predefined categories: “daily”, “3–4 times/week”, “1–2 times/week”, “1–2 times/month” or “almost never”.

For patients with EDS and criteria indicating moderate to severe OSA (loud snoring, witnessed apneas/gasping/choking, hypertension [two out of three criteria]), single-night home sleep apnea testing (HSAT) was performed according to American Academy of Sleep Medicine (AASM) guidelines [35,36]. HSAT included nasal pressure, thoracic and abdominal excursion using respiratory inductance plethysmography (RIP) technology, oxygen saturation, electrocardiogram and body position sensors. Apnea was defined as ≥90% airflow cessation for ≥10 s and hypopnea as ≥30% reduction in airflow accompanied by ≥3% oxygen desaturation [37,38]. The AHI was calculated as the total number of apneas and hypopneas per hour of recording encompassing the habitual sleep period. In patients with severe insomnia, cardiorespiratory comorbidities and other contraindications for HSAT, polysomnography (PSG) was performed [35,36]. Apnea was defined as ≥90% airflow cessation for ≥10 s, hypopnea as ≥30% reduction in airflow accompanied by ≥3% oxygen desaturation from pre-event baseline or the event was associated with an arousal [37,38]. The AHI was calculated as the total number of apneas and hypopneas per hour of sleep. Patients with OSA were subsequently categorized as mild (AHI 5–14.9), moderate (AHI 15–29.9), or severe OSA (AHI ≥ 30).

Clinical variables from 3227 OSA patients’ charts and PSG/HSAT variables were used to create a database, following approval by the Institutional Review Board of “Georgios Papanikolaou” General Hospital of Thessaloniki, Greece (IRB No: 965/290618). Patient data were anonymized to ensure confidentiality. Inclusion criteria required a confirmed OSA diagnosis, determined via PSG or HSAT and full data on the sleep habits questionnaire question about MHs. Patients with primary sleep-related breathing disorders other than OSA or missing critical data were excluded.

ICHD-3 classifies MHs as sleep apnea headaches in OSA patients if two elements from the description below are also present: recurring pain > 15 days in a month or is resolved within 4 h or has a pressing quality, bilateral localization without nausea, photo- or phonophobia [14]. We use the term MHs instead of sleep apnea headache because the sleep habits questionnaire did not require patients to describe accompanying symptoms to their headache, and also, the secondary criterion “disappearance of headache within 72 h after CPAP treatment” was not always met due to missing data on therapeutic interventions. Because questionnaire data did not permit formal ICHD-3 classification, a pragmatic frequency-based definition and categorization was adopted, consistent with previous epidemiological studies [1,39]. Therefore, those patients reporting ≥ 1 MHs per week were classified into the frequent MH group and those with <1 episode were assigned to the control group. The use of this cutoff in the literature and in this study reflects evidence that four or fewer monthly headache days is associated with lower chronification risk, making it a meaningful treatment target as well as a clinical warning sign [40]. As an example, four or more headache days per month also serve as the clinical threshold for initiating migraine prophylaxis [41,42].

Analysis was carried out using SPSS Statistics 20.0 software (IBM Corp, Armonk, NY, USA) following STROBE guidelines for observational studies [43]. Given the large sample size, parametric analyses were considered robust to moderate deviations from normality according to the central limit theorem. Descriptive statistics summarized continuous variables as means ± standard deviations (or median and interquartile range), while categorical variables were reported as absolute and relative frequencies. Between-group comparisons were performed using independent-samples *t*-tests or Mann–Whitney U tests for continuous variables and χ^2^ tests or Fisher’s exact tests, as appropriate. Statistical significance was defined as a two-sided *p*-value < 0.05.

To search for risk factors for MHs and for relations between MHs and other OSA-related parameters, a univariate logistic regression analysis was performed. Complete data on OSA diagnosis and MH frequency were available for all 3227 patients. Missing data across other variables but not strictly PSG-related variables were negligible (N = 54, <2%) and handled by complete-case analysis. REM sleep time and arousal index were inherently only available in those patients that underwent PSG (N = 501) and not HSAT. Additional univariate logistic regression analyses were also performed in women only (N = 880) to identify factors associated with MHs in this subgroup.

Variables that were statistically significant in the univariate logistic regression analyses (*p* < 0.05) for the whole cohort (N = 3227 patients) were entered into the initial multivariable logistic regression model. These included: sex, BMI, waist circumference, GERD, total sleep time, REM sleep time, the AHI, OSA severity category, mean SaO_2_, minimum SaO_2_, time spent with SaO_2_ < 90%, the ODI, arousal index, AIS and ESS scores (15 variables in total). A backward likelihood ratio selection procedure was subsequently applied to obtain the final model. The multivariable analysis was conducted using complete-case data. Because the arousal index was available only in patients who underwent PSG, whereas most patients underwent HSAT, the final multivariable analysis was restricted to the 501 patients who had complete data for this candidate predictor.

Multicollinearity was formally assessed for the variables retained in the final multivariable model (female sex, waist circumference and arousal index) using condition indices, tolerance values and variance inflation factors, using predefined thresholds. Model robustness was evaluated through multiple alternative variable selection procedures (Enter, Forward Conditional, Forward LR, Forward Wald, Backward Conditional, Backward LR, and Backward Wald methods), and internal validation was performed using bootstrap resampling (1000 samples) [34,35,36,37,38,39,40,41,42,43,44,45,46].

BMI and waist circumference were entered simultaneously in the model because they capture different measures of adiposity, especially in relation to OSA. Multiple adult cohorts show waist circumference, BMI, and neck circumference to be independently associated with OSA presence and severity [47,48] and may have distinct pathophysiological associations with headache symptoms [49]. Because both variables were statistically significant in the univariate analyses, both were entered into the initial multivariable model, and their collinearity was also assessed in the initial model.

## 3. Results

The study included 3227 patients diagnosed with OSA who were stratified into two groups based on MH frequency: those experiencing headaches rarely or never (never, 1–2/month, N = 2092) and those reporting MHs more than once per week (everyday, 3–4/week, 1–2/week, N = 1135). Most participants reported headaches “almost never” (861 total), with daily headaches being rare (5 total). Demographic, clinical and sleep study characteristics are presented in Table 1.

Patients with MHs were more frequently female, had higher BMIs and waist circumferences, and more frequently reported snoring, fatigue and OSA burden (all *p* < 0.05). In contrast, no significant differences were observed between groups regarding neck circumference, non-REM sleep duration, central apnea burden, hypertension, coronary heart disease, diabetes mellitus, arrhythmia, OSA severity category, arousal index, or the distribution of diagnostic sleep studies (PSG versus HSAT) (all *p* > 0.05).

After subgrouping on the basis of diagnostic modality, compared with patients diagnosed using HSAT (N = 2726), the PSG subgroup was slightly older and had a smaller waist circumference, but effect sizes were small. However, the two groups did not differ significantly in the prevalence of MHs (38.3% vs. 34.6%, *p* = 0.11), sex distribution, BMI, fatigue, daytime sleepiness, insomnia symptoms, or major comorbidities (Supplementary Table S4), suggesting that the PSG subgroup was broadly comparable to the HSAT cohort with respect to the measured clinical characteristics relevant to MHs, although comparability for unmeasured factors cannot be established.

**Figure 1:**
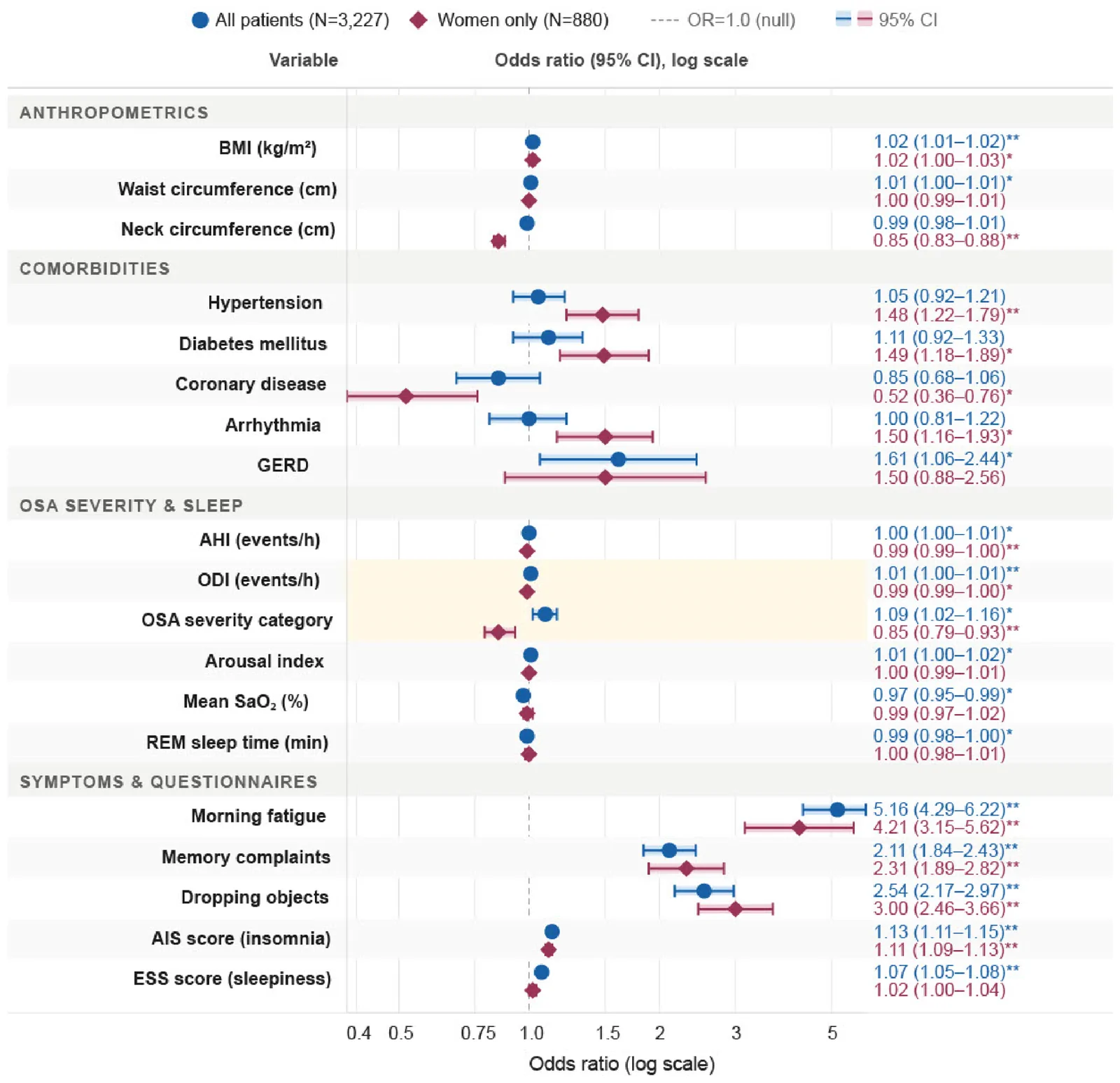
Univariate risk factors for morning headaches: all patients vs. women only. * *p* < 0.05, ** *p* < 0.001, all other: not significant (*p* > 0.05). Yellow shading indicates variables where direction of association reverses between cohorts (both significant). CI—confidence interval; OR—odds ratio; AHI—apnea–hypopnea index; ODI—oxygen desaturation index; AIS—Athens Insomnia Scale; ESS—Epworth Sleepiness Scale; GERD—gastro-esophageal reflux disease. Morning fatigue capped at OR = 5.5 for display purposes.

In univariate logistic regression analyses, several demographic, anthropometric, PSG and clinical variables were associated with the presence of MHs. Female sex was associated with nearly twice the odds of MHs compared with male sex (OR = 1.93, 95% CI 1.67–2.23, *p* < 0.001). Measures of adiposity were also significantly associated with MHs, although the magnitude of these effects was small, as shown in Figure 1. No comorbidities other than GERD were associated with increased odds of MHs (*p* > 0.05). Among sleep study parameters, MHs were associated with indices reflecting sleep-disordered breathing severity and nocturnal hypoxemia, including minimum oxygen saturation (OR = 0.99, 95% CI 0.99–1.00, *p* = 0.027), longer time spent with oxygen saturation below 90% (OR = 1.00, 95% CI 1.00–1.01, *p* = 0.001) and snoring (OR = 2.61, 95% CI 1.62–4.19, *p* < 0.001). However, the oxygenation parameters showed that the effect sizes were minimal and of limited clinical relevance.

MHs were also associated with a broad range of OSA-related symptoms and questionnaire measures. The largest association was observed for morning fatigue but also other daytime symptoms, as shown in Figure 1. Significant associations were also found for nocturnal diuresis (OR = 1.38, 95% CI 1.16–1.63, *p* < 0.001) and choking (OR = 1.91, 95% CI 1.51–2.42, *p* < 0.001), as well as self-reported awakenings (OR = 1.95, 95% CI 1.63–2.32, *p* < 0.001) and awakenings due to choking (OR = 2.41, 95% CI 2.10–2.76, *p* < 0.001).

Additional univariate logistic regression analyses were performed in women only (Figure 1). In this subgroup, several comorbidities demonstrated significant associations, in contrast with the study group as a whole. Among sleep study parameters, MHs were associated with lower indices of milder OSA burden, including shorter mean (OR = 0.97, 95% CI: 0.96–0.99, *p* < 0.001) and max apnea duration (OR = 0.99, 95% CI: 0.99–1.00, *p* < 0.001), as shown in Figure 1, but effect sizes were small. MHs in women were also associated with OSA-related symptoms and functional parameters (Supplementary Table S3), such as nocturnal awakenings (OR = 2.10, 95% CI 1.60–2.76, *p* < 0.001).

Variables that were significant in the univariate analyses were entered into a multivariable logistic regression model, restricted to the subgroup that was initially diagnosed through PSG, using backward likelihood ratio selection. In the final model, female sex emerged as the strongest independent predictor of MHs, with women having more than fourfold higher odds of MHs than men (OR = 4.23, 95% CI 1.39–12.85, *p* = 0.011). Waist circumference remained independently associated with MHs (OR = 1.03, 95% CI 1.01–1.06, *p* = 0.019), as did the arousal index (OR = 1.05, 95% CI 1.01–1.08, *p* = 0.004). No other demographic, PSG or OSA severity variables retained statistical significance after adjustment in this subgroup.

The standard error of the regression coefficient for female sex remained relatively small (approximately 0.56). Bootstrap resampling produced comparable estimates, with a 95% CI for the regression coefficient remaining statistically significant (0.516–4.310) and an observed bias (0.507) smaller than the corresponding standard error (1.153). Condition indices derived from the collinearity diagnostics were 12.722 for waist circumference and arousal index, 5.864 for female sex and waist circumference, and 5.841 for female sex and arousal index, below commonly accepted thresholds indicating problematic collinearity [44]. Furthermore, tolerance values were approximately 1.0 and variance inflation factors were approximately 1.0 for all retained variables in the final model, indicating negligible multicollinearity [45,46]. In the initial multivariable model, the condition index was 23.1, VIF was 2.62 and tolerance was 0.38 for BMI and waist circumference, collectively indicating that this correlation did not constitute problematic multicollinearity [44,45,46].

## 4. Discussion

In this large retrospective cohort of 3227 patients with OSA, frequent MHs were common, as 1136 out of 3227 patients (35.2%) reported headaches more than once per week, a finding consistent with the literature [2,3,10,11]. This prevalence is higher than would be expected in the general population [1] and in other sleep disorders [12] and supports the notion that MHs are a common symptom among patients referred for OSA evaluation [50]. Previous studies in OSA cohorts similarly found that headache was more strongly associated with clinical symptoms such as fatigue rather than with conventional measures of OSA severity, including the AHI, supporting the concept that MHs may reflect broader disease burden rather than respiratory severity alone [51]. However, because the sample consists entirely of sleep clinic patients, the prevalence should not be interpreted as representative of all OSA patients in the community, as referral populations are enriched for symptomatic patients. Daily headaches were very rare (five patients), suggesting that the burden is driven primarily by intermittent but recurrent headaches rather than chronic daily headache.

Despite MHs having been recognized as a symptom of OSA syndrome, the ICHD-3 explicitly notes that MHs are a non-specific symptom, and one of the principal considerations when interpreting the present findings is the distinction between MHs as assessed in this study and the ICHD-3 diagnosis of sleep apnea headache [14]. In the present study, headache assessment was based on self-reported frequency of headache upon awakening, without detailed characterization of headache phenotype, associated symptoms, duration, or response to CPAP therapy. Consequently, formal classification of sleep apnea headache was not possible, and the term MH was deliberately adopted to reflect a broader clinical construct. Studies in habitual snorers similarly suggest that MHs represent a heterogeneous symptom, with many headaches not fulfilling formal criteria for sleep apnea headache, further supporting the distinction between self-reported MHs and a specific OSA-related headache disorder [52,53]. This distinction is particularly relevant because the ICHD-3 also recognizes that MHs occur across diverse primary and secondary headache disorders, as well as primary sleep disorders such as periodic limb movements, complicating mechanistic attribution [14]. Furthermore, headaches of non-OSA etiology and sleep disorders (including OSA) exhibit significant comorbidity. Studies demonstrate a high prevalence of OSA among patients with cluster headache [54] and migraine [27]. Notably, 60–70% of migraine patients have reported MHs, with OSA possibly being an underlying factor [27,28]. Despite the conditions being quite common in the general population independently, their co-occurrence has been attributed to possibly shared pathophysiological mechanisms [7,28,55].

Research has predominantly focused on the role of nocturnal respiratory disturbance characteristics of OSA, including heavy snoring, intermittent hypoxia, hypercapnia and consequent blood pressure fluctuations, in provoking headaches that persist upon awakening [2]. The consistent observation that effective OSA treatment, particularly CPAP therapy, resolves or significantly ameliorates MHs in affected patients provides compelling evidence for a causal relationship between OSA pathophysiology and headache generation [3,19]. Effective PAP therapy, by mitigating hypoxemia and hypercapnia, typically resolves or significantly improves MHs in the majority of patients, with studies reporting resolution rates of 72.4% on the first treatment day, rising to 92.1% after one month of consistent use [3]. Nevertheless, the ICHD-3 acknowledges persistent uncertainty regarding whether hypoxia, hypercapnia, sleep disturbance, or a combination thereof constitutes the primary driver of sleep apnea headache [14]. Indeed, while the elevated AHI, ODI and desaturation severity indices in our MH group align with the hypothesis that intermittent hypoxia may contribute to headache pathophysiology [24] as in sleep apnea headache [14], the predictive significance of these metrics attenuated in the multivariable model once arousal index and patient characteristics were considered. This dissociation may suggest that MH etiology in OSA may be more closely related to sleep continuity disruption that could possibly lead to sympathetic activation [56] than to respiratory severity indices. These findings could align with evidence that microarousals and poor sleep perception may activate trigeminovascular pathways or cortical hyperexcitability, precipitating headaches [57,58] and challenge the conventional emphasis on the AHI as the principal determinant of OSA-related morbidity. These mechanisms often overshadow hypoxemia’s direct effects, explaining why CPAP improved headaches in other studies, even when oxygen parameters remained suboptimal [39] and also align with the notion that hypoxemia is clinically relevant among physiologically susceptible patients [2,21,27]. However, the attenuation of respiratory indices alongside the persistence of arousal index as a predictor is equally compatible with sleep fragmentation aggravating an underlying, unrecognized primary headache disorder such as migraine, as these mechanisms also serve as triggers for these diseases [28,29]. In the absence of headache phenotyping and medication history data in the present cohort, these two explanations cannot be distinguished. Finally, the dissociation between comorbidities and MHs in this study could support the hypothesis that OSA-related headaches are driven by sleep disturbances and not, for example, by hypertension or metabolic syndrome, as in primary headache disorders [59,60,61].

An interesting finding was that waist circumference remained independently associated with frequent MHs after multivariable adjustment, whereas BMI did not, suggesting that central adiposity may be more relevant than overall obesity in this context. This finding is consistent with evidence indicating that measures of abdominal fat distribution often provide additional information beyond BMI in OSA, and may correlate more closely with disease severity and its metabolic consequences [62,63]. A similar pattern has been described in headache disorders, particularly migraine, where larger waist circumference and related indices have been associated with greater headache frequency and severity, although the independent contribution of central obesity remains inconsistent across studies [64,65,66]. Nevertheless, the magnitude of the association observed in our study was modest, and caution is warranted in attributing a causal role to central obesity. Most available evidence is cross-sectional, studies specifically addressing MHs are lacking, and associations between waist-based measures and headache in other studies appear to be influenced by age, sex and behavioral factors, with several investigations reporting attenuation or loss of significance after adjustment for lifestyle and clinical covariates [67,68,69]. Therefore, our findings should be interpreted as suggesting that central adiposity may represent a marker of a broader metabolic and inflammatory phenotype associated with recurrent MHs, rather than establishing abdominal obesity as an independent pathogenic factor.

Finally, female sex emerged as the strongest independent predictor of frequent MHs, with women exhibiting more than fourfold higher odds of headache after adjustment, for the PSG subgroup. This finding was expected, as women classically present with atypical OSA symptomatology compared to men, including MHs [29], and also the female predominance of migraine and, in many datasets, tension-type headaches [70,71]. Univariate analyses in our study, restricted to all women, irrespective of diagnostic pathway, revealed a distinct pattern in which MHs were associated predominantly with symptom burden rather than with conventional markers of OSA severity. Women with frequent MHs reported substantially higher rates of fatigue, nocturnal awakenings, awakenings due to choking, memory complaints and impaired motor function, whereas respiratory indices paradoxically suggested a milder OSA phenotype, characterized by lower AHI and ODI values, lower OSA severity categories and shorter mean and maximum apnea durations. These findings highlight the limitations of relying solely on the AHI to assess OSA-related morbidity in this population. Previous studies have shown that women with OSA frequently present with less typical symptoms and are more likely to report fatigue, insomnia, unrefreshing sleep and MHs rather than classic manifestations such as loud snoring and witnessed apneas, contributing to delayed diagnosis and under-recognition of disease severity [30,72]. Moreover, women appear to experience greater morbidity and mortality for a given degree of OSA severity [29], suggesting that physiological consequences may not be adequately captured by conventional respiratory metrics. Several mechanisms may contribute to this phenotype. Experimental and clinical evidence suggests that sleep fragmentation exerts a greater impact on pain processing in women, while fluctuations in sex hormones could theoretically further increase susceptibility to headache and alter sleep architecture [24,73,74]. At the same time, sex differences in symptom awareness and reporting, together with the lower likelihood of bed partners reporting snoring or witnessed apneas in women, may contribute to apparent discrepancies between subjective symptom burden and objective OSA severity [29]. Clinically, these findings could suggest that recurrent MHs in women should prompt consideration of OSA even in the presence of relatively mild respiratory disturbance and emphasize the need for sex-specific approaches that extend beyond AHI-based definitions of disease severity.

## 5. Limitations and Conclusions

The present study has several important limitations that should be considered when interpreting the findings. First, its retrospective, single-center design limits causal inference and may introduce referral bias, as the cohort consisted exclusively of symptomatic patients referred to a sleep clinic and therefore may not be representative of the general OSA population. MHs were defined pragmatically according to frequency of headaches upon awakening and not according to the full ICHD-3 criteria for sleep apnea headaches, because information regarding headache phenotype, duration, associated symptoms and response to CPAP therapy was unavailable. Consequently, headaches from other primary or secondary causes could not be excluded. In particular, we could not adjust for a history of migraine or tension-type headache, nor for migraine-specific medication use. Although the overall sample was large, the multivariable analysis was necessarily restricted to the subgroup undergoing PSG (N = 501), since arousal index and REM sleep data were unavailable in patients evaluated with HSAT, raising the possibility of selection bias and limiting the generalizability of the multivariable findings, particularly the identification of arousal index as an independent predictor of MHs. While PSG and HSAT subgroups were broadly comparable with respect to the principal clinical characteristics relevant to MHs, unmeasured differences related to referral for PSG rather than HSAT cannot be excluded. We also acknowledge that the wider confidence interval observed for female sex in the multivariable model is partly attributable to the reduced sample size available for complete-case analysis (N = 501), compared with the full cohort used in the univariate analyses (N = 3227). Nevertheless, multicollinearity was formally assessed and found to be negligible and similar estimates were obtained using alternative variable selection approaches. Furthermore, complete-case analysis was used for missing variables; no information was available regarding medication use, psychiatric disorders, analgesic consumption, hormonal status, or pre-existing headache syndromes; and residual confounding could not be excluded. Finally, the cross-sectional nature of the analysis precludes conclusions regarding whether sleep fragmentation or obesity are causally involved in headache generation.

In conclusion, frequent MHs were common among patients with OSA, affecting approximately one-third of this large clinical cohort. Although patients with MHs exhibited greater nocturnal hypoxemia and more severe sleep-disordered breathing on univariate analysis, these indices lost significance after adjustment, whereas female sex, central adiposity and sleep fragmentation, reflected by the arousal index, remained independently associated with headache occurrence. These findings suggest that disrupted sleep continuity may play a more important role in the pathophysiology of OSA-related MHs than conventional respiratory severity measures such as the AHI. In addition, in women, headache burden was associated more closely with symptoms and sleep disruption than with traditional markers of OSA severity. These results point to the limitations of relying solely on the AHI to assess disease burden and support a more comprehensive and sex-specific approach to the evaluation of OSA patients presenting with recurrent morning headaches. Prospective studies incorporating formal headache classification and treatment outcomes are warranted to clarify the mechanisms underlying this association and determine whether targeting sleep fragmentation improves headache symptoms.

## Figures and Tables

**Table 1:** Demographic and clinical characteristics by morning headache status.

	MH < 1/Week (N = 2092)	MH ≥ 1/Week (N = 1135)	Effect Size	*p* (Value)
Age, years	58.76 ± 13.03	58.02 ± 12.65	0.057	0.11
Male sex N, %	1614 (77.15)	733 (64.58)	0.278	<0.001
Female sex N, %	478 (22.84)	402 (35.41)	0.278	<0.001
BMI, kg/m^2^	32.61 ± 8.49	33.67 ± 9.78	0.118	0.001
Waist circumference, cm	113.78 ± 17.15	115.97 ± 16.98	0.128	0.009
Snoring, %	95.60	98.50	0.177	<0.001
Fatigue, %	59.65	88.20	0.675	<0.001
GERD, %	1.86	3.08	0.079	0.036
PSG N, %	309 (14.77)	192 (16.92)	0.059	0.110
HSAT N, %	1783 (85.22)	943 (83.09)	0.059	0.110
Total sleep time, minutes	262.84 ± 80.70	227.79 ± 76.82	0.442	0.003
REM sleep time, minutes	57.08 ± 36.03	40.76 ± 32.62	0.468	0.004
AHI, events/h	36.67 ± 22.76	38.90 ± 23.94	0.096	0.008
Mean SaO_2_ (%)	91.63 ± 3.69	91.26 ± 3.47	0.102	0.005
Minimum SaO_2_ (%)	77.72 ± 9.28	77.04 ± 9.67	0.072	0.040
Time Sat < 90%, minutes	10.3 (1.9–35.4)	15 (2.2–42.5)	0.443	0.006
ODI, events/h	35.98 ± 23.27	38.81 ± 24.58	0.119	0.001
ESS score	9.34 ± 4.48	10.67 ± 4.70	0.292	<0.001
AIS score	7.80 ± 5.19	11.39 ± 5.21	0.691	<0.001

*Values are mean ± SD unless otherwise indicated. Time with saturation under 90% in minutes is summarized as median (IQR) because of right-skewed distribution. For effect sizes, absolute Cohen’s d for continuous variables and Cohen’s h for categorical variables are reported, except for time with saturation under 90%, for which Cliff’s delta is reported. BMI—body mass index; GERD—gastro-esophageal reflux disease; PSG—polysomnography; HSAT—home sleep-apnea testing; AHI—apnea–hypopnea index; SaO_2_—oxygen saturation; Time Sat < 90%—time spent with oxygen saturation below 90%; ODI—oxygen desaturation index; ESS—Epworth Sleepiness Scale; AIS—Athens Insomnia Scale.*

## Data Availability

The raw data supporting the conclusions of this article will be made available by the authors on request.

## Supplementary Materials

The following supporting information can be downloaded at: https://www.mdpi.com/article/10.3390/life16081239/s1, Table S1: Univariate logistic regression analysis for morning headaches—N = 3227 patients; Table S2: Multivariable logistic regression analysis of the risk factors for morning headaches—final step of the backward likelihood ratio procedure; Table S3: Univariate logistic regression analysis for morning headaches—N = 880 women; Table S4: Stratification based on diagnostic modality.

## Abbreviations

The following abbreviations are used in this manuscript:

AASM	American Academy of Sleep Medicine
AHI	Apnea–Hypopnea Index
AIS	Athens Insomnia Scale
BMI	Body Mass Index
CI	Confidence Interval
CPAP	Continuous Positive Airway Pressure
EDS	Excessive Daytime Sleepiness
ESS	Epworth Sleepiness Scale
GERD	Gastroesophageal Reflux Disease
HSAT	Home Sleep Apnea Testing
ICHD	International Classification of Headache Disorders
ICHD-3	International Classification of Headache Disorders, Third Edition
IQR	Interquartile Range
IRB	Institutional Review Board
LR	Likelihood Ratio
MH	Morning Headache
ODI	Oxygen Desaturation Index
OR	Odds Ratio
OSA	Obstructive Sleep Apnea
PSG	Polysomnography
REM	Rapid Eye Movement
RIP	Respiratory Inductance Plethysmography
SaO_2_	Arterial Oxygen Saturation
SD	Standard Deviation
SPSS	Statistical Package for the Social Sciences
STROBE	Strengthening the Reporting of Observational Studies in Epidemiology
