# Supplementary material for: Morning Headaches as a Specific Obstructive Sleep Apnea Characteristic in a Large Sleep Clinic Population
Source: Life (Basel). 2026 Jul 27;16(8):1239. doi: 10.3390/life16081239 (PMC13514924; doi:10.3390/life16081239)
Supplement: Supplementary file 1 [file life-16-01239-s001.zip › life-4423144-supplementary.pdf]

E-Supplement

Supplementary Table S1. Univariate logistic regression analysis for morning headaches – N=3227 patients

Variable	OR (95% C.I.)	p (value)
Risk factors for morning headaches		
Age (years)	1.00 (0.99 – 1.00)	0.09
Sex (female)	1.93 (1.67 – 2.23)	**<0.001**
BMI (Kg/m^2)	1.02 (1.01 – 1.02)	**<0.001**
Neck circumference (cm)	0.99 (0.98 – 1.01)	0.23
Waist circumference (cm)	1.01 (1.00 – 1.01)	**0.004**
Smoking (packyears)	1.00 (1.00 – 1.00)	0.59
Hypertension (yes)	1.05 (0.92 – 1.21)	0.47
Diabetes mellitus (yes)	1.11 (0.92 – 1.33)	0.27
Coronary disease (yes)	0.85 (0.68 – 1.06)	0.15
Arrythmia (yes)	1.00 (0.81 – 1.22)	0.96
Dyslipidemia (yes)	0.99 (0.82 – 1.18)	0.88
Pulmonary disease (yes)	0.95 (0.75 – 1.21)	0.69
GERD (yes)	1.61 (1.06 – 2.44)	**0.025**
Total sleep time (minutes)	1.00 (0.99 – 1.00)	**0.003**
REM sleep time (minutes)	0.99 (0.98 – 1.00)	**0.018**
Non-REM sleep time (minutes)	1.00 (0.99 – 1.00)	0.05
AHI (total score)	1.00 (1.00 – 1.01)	**0.002**
Central apneas (total score)	1.01 (0.99 – 1.03)	0.42
OSA (severity category)	1.09 (1.02 – 1.16)	**0.009**
Mean SaO_2 (%)	0.97 (0.95 – 0.99)	**0.004**
Minimum SaO_2 (%)	0.99 (0.99 – 1.00)	**0.027**
Time with SaO_2 below 90% (minutes)	1.00 (1.00 – 1.01)	**0.001**
ODI (total score)	1.01 (1.00 – 1.01)	**<0.001**
Arousal index (total score)	1.01 (1.00 – 1.02)	**0.003**
Mean apnea duration (seconds)	0.99 (0.98 – 1.00)	0.12
Maximum apnea duration (seconds)	1.00 (1.00 – 1.00)	0.93
Athens' insomnia scale (total score)	1.13 (1.11 – 1.15)	**<0.001**
Epworth sleepiness scale (total score)	1.07 (1.05 – 1.08)	**<0.001**
Morning fatigue (yes)	5.16 (4.29 – 6.22)	**<0.001**
Nocturnal diuresis (yes)	1.38 (1.16 – 1.63)	**<0.001**
Night awakenings (yes)	1.95 (1.63 – 2.32)	**<0.001**
Snoring (yes)	2.61 (1.62 – 4.19)	**<0.001**
Chocking (yes)	1.91 (1.51 – 2.42)	**<0.001**
Awakenings due to choking (yes)	2.41 (2.10 – 2.76)	**<0.001**
Memory loss (yes)	2.11 (1.84 – 2.43)	**<0.001**
Dropping things from hands (yes)	2.54 (2.17 – 2.97)	**<0.001**

Supplementary Table S2. Multivariable logistic regression analysis of the risk factors for morning headaches - final step of the backward likelihood-ratio procedure

Variable	OR (95% C.I.)	p (value)
Sex (female)	4.23 (1.39 – 12.85)	**0.011**
BMI (Kg/m^2)	1.00 (0.88 – 1.13)	0.99

Waist circumference (cm)	1.03 (1.01 – 1.06)	**0.019**
GERD (yes)	n/a	1.00
Total sleep time (minutes)	1.00 (0.99 – 1.00)	0.29
REM sleep time (minutes)	1.00 (0.98 – 1.01)	0.70
AHI (total score)	1.01 (0.95 – 1.06)	0.86
OSA (severity)	0.93 (0.58 – 1.47)	0.74
Mean SaO_2 (%)	1.02 (0.91 – 1.14)	0.73
Minimum SaO_2 (%)	1.00 (0.93 – 1.07)	0.93
Time with SaO_2 below 90% (minutes)	1.00 (0.99 – 1.02)	0.79
Time with SaO_2 below 85% (minutes)	1.01 (0.98 – 1.04)	0.54
ODI (total score)	1.00 (0.97 – 1.03)	0.95
Arousal index (total score)	1.05 (1.01 – 1.08)	**0.004**

Supplementary Table S3. Univariate logistic regression analysis for morning headaches – N=880 women

Variable	OR (95% C.I.)	p (value)
Risk factors for morning headaches in women		
Age (years)	1.01 (1.00 – 1.02)	0.06
BMI (Kg/m^2)	1.02 (1.00 – 1.03)	**0.008**
Neck circumference (cm)	0.85 (0.83 – 0.88)	**<0.001**
Waist circumference (cm)	1.00 (0.99 – 1.01)	0.75
Smoking (packyears)	0.99 (0.98 – 0.99)	**<0.001**
Hypertension (yes)	1.48 (1.22 – 1.79)	**<0.001**
Diabetes mellitus (yes)	1.49 (1.18 – 1.89)	**0.001**
Coronary disease (yes)	0.52 (0.36 – 0.76)	**0.001**
Arrythmia (yes)	1.50 (1.16 – 1.93)	**0.002**
Dyslipidemia (yes)	1.20 (0.94 – 1.53)	0.14
Pulmonary disease (yes)	1.06 (0.76 – 1.47)	0.74
GERD (yes)	1.50 (0.88 – 2.56)	0.14
Total sleep time (minutes)	1.00 (0.99 – 1.00)	0.25
REM sleep time (minutes)	1.00 (0.98 – 1.01)	0.52
Non-REM sleep time (minutes)	1.00 (0.99 – 1.01)	0.64
AHI (total score)	0.99 (0.99 – 1.00)	**<0.001**
Central apneas (total score)	0.94 (0.89 – 1.00)	**0.049**
OSA (severity)	0.85 (0.79 – 0.93)	**<0.001**
Mean SaO_2 (%)	0.99 (0.97 – 1.02)	0.60
Minimum SaO_2 (%)	1.01 (1.00 – 1.02)	0.31
Time with SaO_2 below 90% (minutes)	1.00 (1.00 – 1.00)	0.64
Time with SaO_2 below 85% (minutes)	1.00 (0.99 – 1.01)	0.60
ODI (total score)	0.99 (0.99 – 1.00)	**0.004**
Arousal index (total score)	1.00 (0.99 – 1.01)	0.80
Mean apnea duration (seconds)	0.97 (0.96 – 0.99)	**<0.001**
Maximum apnea duration (seconds)	0.99 (0.99 – 1.00)	**<0.001**
Athens’ insomnia scale (total score)	1.11 (1.09 – 1.13)	**<0.001**
Epworth sleepiness scale (total score)	1.02 (1.00 – 1.04)	0.08
Other manifestations of OSA that coexist with morning headaches in women		
Morning fatigue (yes)	4.21 (3.15 – 5.62)	**<0.001**
Nocturnal diuresis (yes)	1.35 (1.05 – 1.72)	**0.019**
Night awakenings (yes)	2.10 (1.60 – 2.76)	**<0.001**

Snoring (yes)	1.43 (0.78 – 2.61)	0.25
Loud snoring (yes)	1.09 (0.61 – 1.97)	0.77
Chocking (yes)	1.25 (0.91 – 1.72)	0.16
Awakenings due to choking (yes)	2.01 (1.66 – 2.44)	**<0.001**
Memory loss (yes)	2.31 (1.89 – 2.82)	**<0.001**
Dropping things from hands (yes)	3.00 (2.46 – 3.66)	**<0.001**

Supplementary Table S4: Stratification based on diagnostic modality

	HSAT (N= 2726)	PSG (N= 501)	Effect size	p (value)
Age, years	58.26 ± 12.70	59.81 ± 13.89	0.120	**0.014**
Male sex N, %	1975 (72.45)	372 (74.25)	0.041	0.437
Female sex N, %	751 (27.54)	129 (25.75)	0.041	0.437
BMI, kg/m²	33.07 ± 8.86	32.50 ± 9.60	0.063	0.193
Waist circumference, cm	115.04 ± 17.15	112.08 ± 16.84	0.172	**0.006**
Snoring, %	96.88	95.41	0.047	0.367
Fatigue, %	69.92	68.66	0.027	0.61
GERD, %	2.23	2.19	0.007	0.873
Hypertension, %	43.69	42.51	0.024	0.661
Diabetes, %	16.76	18.96	0.057	0.256
Coronary Disease, %	11.41	12.97	0.048	0.353
Arrhythmias, %	13.35	12.17	0.035	0.519
MH <1/week N, %	1783 (65.41)	309 (61.67)	0.059	0.110
MH ≥1/week N, %	943 (34.58)	192 (38.32)	0.059	0.110
ESS score	9.81 ± 4.56	9.79 ± 4.82	0.003	0.949
AIS score	9.03 ± 5.45	8.67 ± 5.55	0.066	0.245

** Values are mean ± SD unless otherwise indicated. Time with saturation under 90% in minutes is summarized as median (IQR) because of right-skewed distribution. For effect sizes, absolute Cohen's d for continuous variables and Cohen's h for categorical variables are reported, except for Time with saturation under 90%, for which Cliff's delta is reported. BMI = body mass index; GERD = gastro-esophageal reflux disease; PSG = polysomnography; HSAT = home sleep-apnea testing; MH = morning headaches; AHI = apnea-hypopnea index; SaO2 = oxygen saturation; Time Sat<90% = time spent with oxygen saturation below 90%; ODI = Oxygen Desaturation Index; ESS = Epworth Sleepiness Scale; AIS = Athens Insomnia Scale*
